# Safety and efficacy of long term asfotase alfa treatment in childhood hypophosphatasia

**DOI:** 10.1186/s13052-025-01883-2

**Published:** 2025-03-20

**Authors:** Debora Mariarita d’Angelo, Federico Lauriola, Luisa Silvestrini, Luigia Cinque, Marco Castori, Giulia Di Donato, Armando Di Ludovico, Saverio La Bella, Francesco Chiarelli, Cosimo Giannini, Luciana Breda

**Affiliations:** 1 Department of Pediatrics, University of Chieti-Pescara “G. D’Annunzio”, Chieti, Italy; 2 Division of Medical Genetics, IRCCS “Casa Sollievo Della Sofferenza”, San Giovanni Rotondo, Italy

**Keywords:** Hypophosphatasia, X-linked hypophosphatasia, Asfotase alpha, Genu valgus, Limping, Alkaline phosphatase

## Abstract

**Background:**

Hypophosphatasia (HPP) is a rare inherited disorder characterized by a deficiency of tissue-non-specific alkaline phosphatase (TNSALP) due to loss-of-function variants of the *ALPL* gene. HPP is characterized by an extremely variable age of onset and clinical presentation, largely depending on the type of genetic disruption. Childhood HPP commonly presents with skeletal deformities, bone fragility, precocious tooth loss, muscle weakness and sometimes neurological implications. Laboratory tests usually document low levels of alkaline phosphatase (ALP), and radiologic investigations show peculiar bone abnormalities. Treatment with human recombinant TNSALP (asfotase alpha, Strensiq^®^), available since 2015, is associated with a sudden improvement and a good safety profile.

**Case presentation:**

A previously healthy 15-month-old girl presented with progressive “genu valgus” and sudden limping. The patient was diagnosed with childhood HPP due to the presence of two *ALPL* variants, never described in compound heterozygosity: a missense variant c.571G > A, p.(Glu191Lys), and a frameshift deletion c.963delG; p.(Lys322Argfs*44), both classified as pathogenetic. The child was promptly treated with asfotase alpha, and good improvement was quickly obtained. Efficacy, safety, and good tolerance persisted after a long-term follow-up of 6 years.

**Conclusions:**

Pediatricians should consider HPP in children presenting with a suggestive clinical phenotype. Calcium-phosphorus metabolism, ALP, and vitamin B6 should always be investigated in suspected cases. Moreover, asfotase alfa represents a safe, well-tolerated, and effective drug in children with HPP.

## Background

**Fig. 1:**
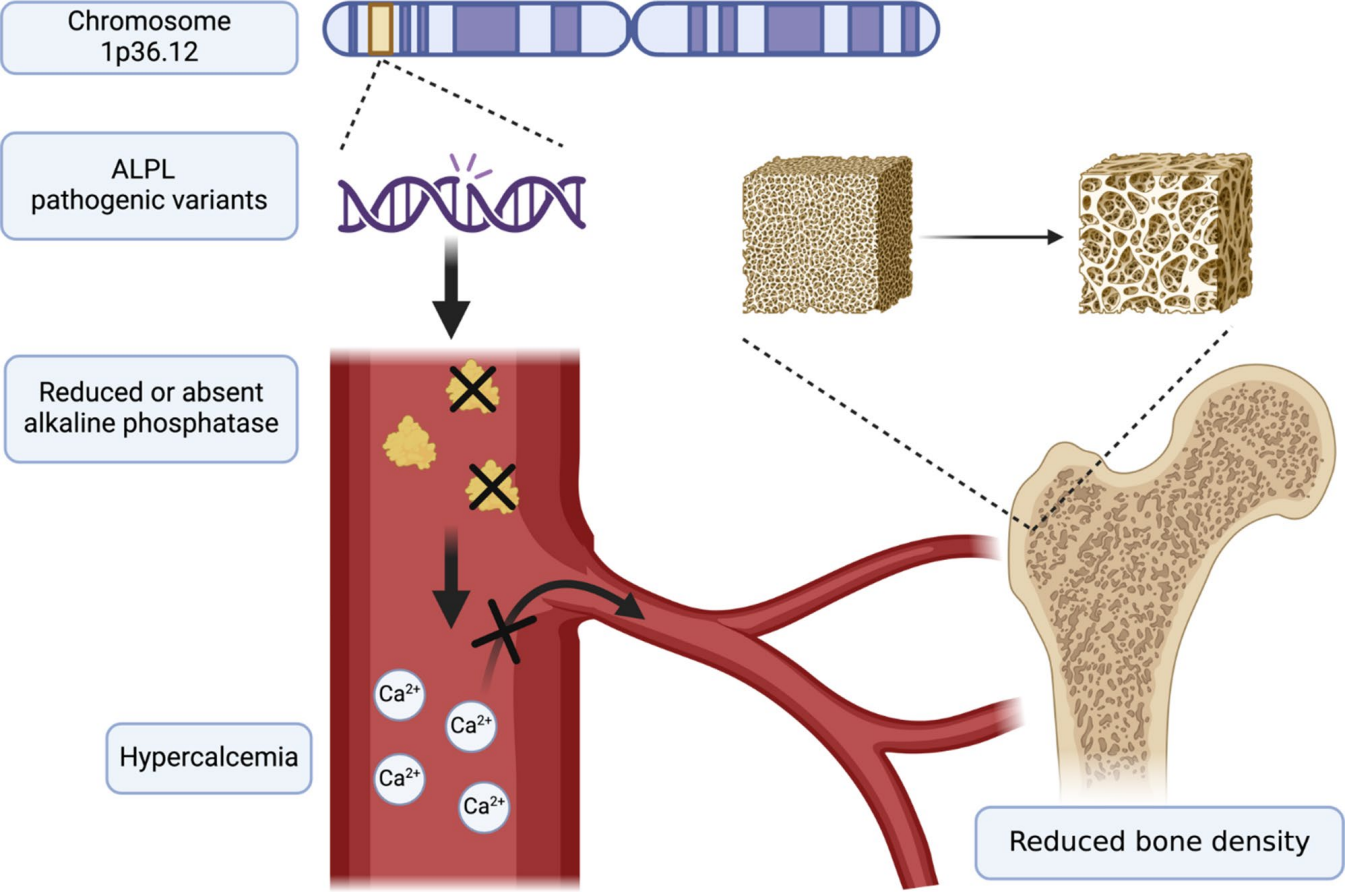
Pathogenic variants of the *ALPL* gene (1p36.12) cause low or absent serum alkaline phosphatases (ALPs). A deficiency of ALP is responsible for elevated pyridoxal-5’-phosphate levels, hypercalcemia, and ineffectiveness in bone mineralization, which leads to bone fragility and reduced density, rickets, and osteomalacia (*created with BioRender.com*)

Hypophosphatasia (HPP) is a rare inherited metabolic disorder caused by the low activity of the enzyme tissue-non-specific alkaline phosphatase (TNSALP) due to loss-of-function pathogenic variants of the *ALPL* gene (1p36.12) [1, 2]. Alkaline phosphatases (ALPs), especially TNSALP, play a crucial role in hydrolyzing extracellular inorganic pyrophosphate, an inhibitor of bone mineralization, to inorganic phosphate, an important component of hydroxyapatite crystals [3]. Additionally, ALP converts extracellular pyridoxal-5’-phosphate (PLP, the major active form of vitamin B6) into pyridoxal, a critical cofactor for the synthesis of various enzymes implicated in the metabolism of some neurotransmitters, such as gamma-aminobutyric acid [2]. The main clinical manifestations of HPP in children are growth failure, skeletal abnormalities, bone fragility due to rickets and osteomalacia, premature deciduous tooth loss, nephrocalcinosis and chondrocalcinosis due to hypercalcemia and hypercalciuria, and neurological involvement with irritability and pyridoxine-responsive seizures (Fig. 1) [1–4]. The continuous characterization of new HPP cases at different ages and the growing discovery of new *ALPL* variants have allowed experts to consider the disease as highly complex, presenting a continuous spectrum of systemic clinical findings with an incomplete genotype-phenotype correlation [1]. The human recombinant TNSALP (asfotase alpha, Strensiq^®^) has been available since 2015, mostly being well tolerated and very effective [3, 4]. However, a small number of pediatric patients treated with asfotase alpha is reported in the literature, with no accurate data on long-term outcomes. We describe the case of a 15-month-old girl with sudden limping and a severe and progressive bilateral “genu valgus” from the acquisition of the standing position related to a severe form of childhood HPP, successfully treated with asfotase alpha with a long-term follow-up of 6 years.

### Case presentation

**Fig. 2:**
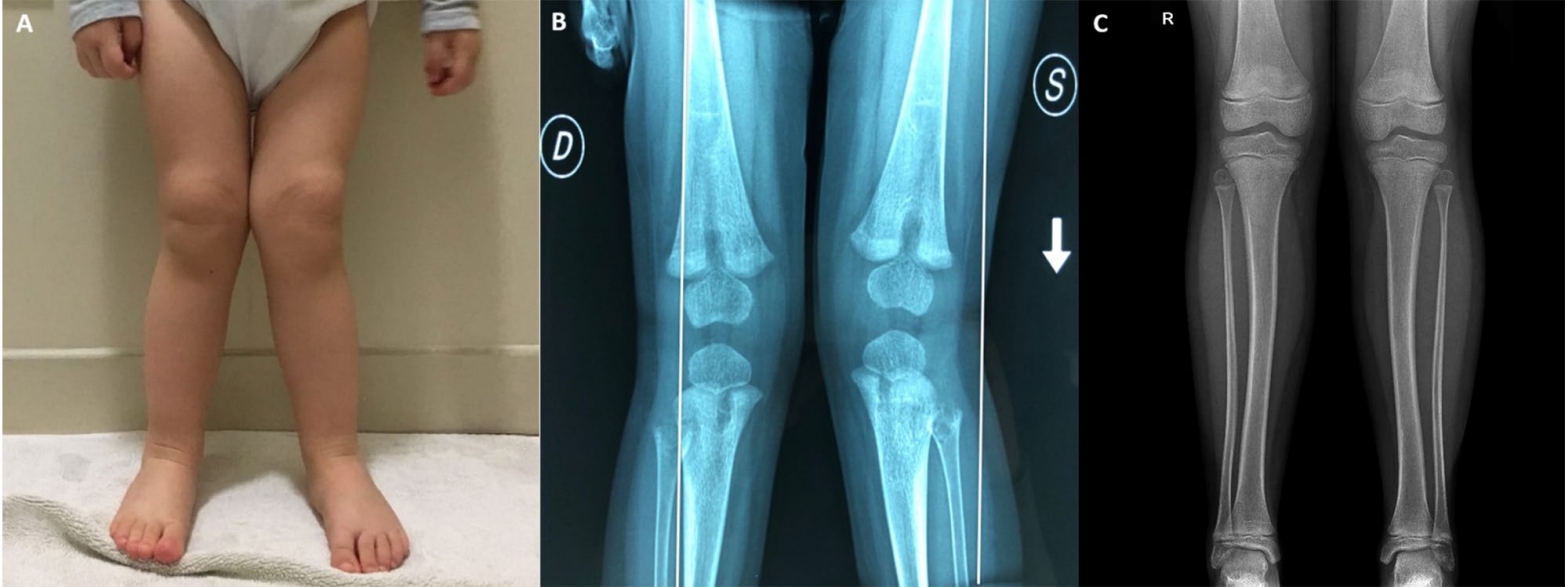
Childhood hypophosphatasia: clinical and radiologic features. (**A**) A 15-month-old girl presented with limping and genu valgus; (**B**) At admission, bilateral knee x-ray documented the bilateral presence of typical central metaphyseal radiolucencies, together with various osteolytic areas with sclerotic margins at the peroneal head and at the proximal tibial metaepiphysis, without any interruptions of the adherent cortical; (**C**) After 6 years of treatment with asfotase alpha, a follow-up radiographic examination showed a “bowing” appearance of the tibial diaphysis without focal morphostructural changes

**Fig. 3:**
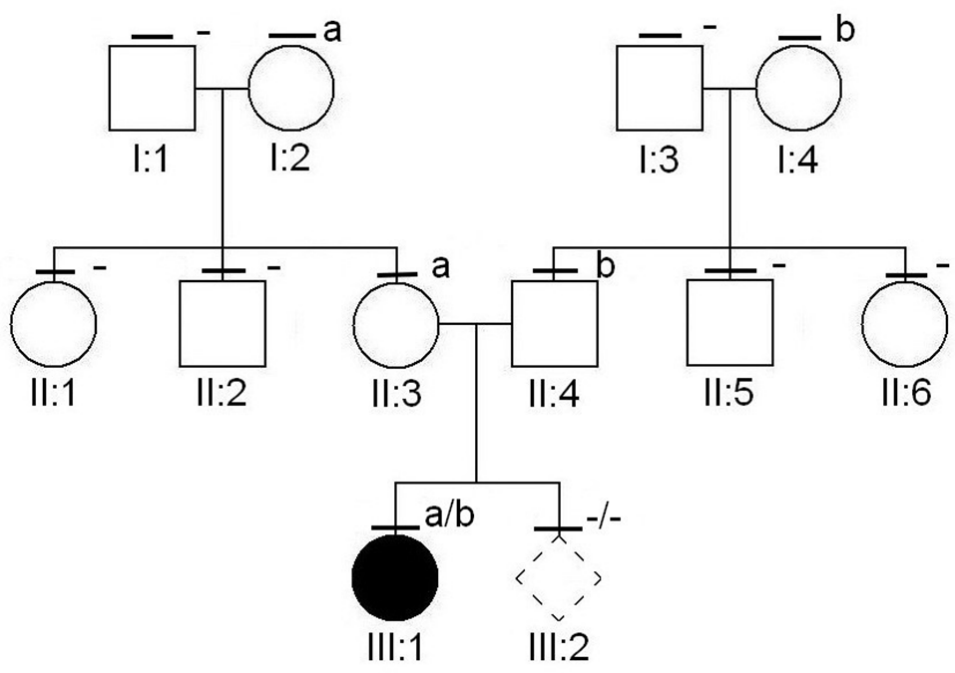
Pedigree of the family under study; dark symbol = clinically affected; the dash above the symbol indicates that the subject undergone the segregation analysis; a = c.571G > A, p.(Glu191Lys); b = c.963delG, p.(Lys322Argfs*44)

A 15-month-old girl presented to our attention due to a progressive bilateral “genu valgus” from 3 months, recently associated with limping (Fig. 2A). Her medical history was unremarkable, with a growth rate between the 25—50th percentile. The child was born at term from unrelated parents, and there were no notable familial history issues. At physical examinations, neither joint swelling nor other signs of arthritis were noted, and there was no history of fever. The complete blood count, kidney and liver indices, and thyroid function were all normal. Screening for celiac disease and C-reactive protein were negative. Serum phosphorus was elevated, and serum calcium, vitamin D, and parathyroid hormone levels were within the normal range. Interestingly, serum ALP was found below the reference range for age and sex (20 U/L; normal values: 150—700 U/L), while PLP was significantly increased (Table 1). A bilateral knee x-ray confirmed the bilateral valgus deformity and documented the bilateral presence of typical central metaphyseal radiolucencies, together with various osteolytic areas with sclerotic margins at the peroneal head and at the proximal tibial metaepiphysis (Fig. 2B). Taken together, these findings were compatible with a diagnosis of childhood HPP. Therefore, genetic investigations were conducted using the Sanger sequencing method and identified two *ALPL* variants in compound heterozygosity: a missense variant [c.571G > A, p.(Glu191Lys)] in exon 6 and a frameshift deletion [c.963delG; p.(Lys322Argfs*44)] in exon 9, the former inherited from the mother and the latter from the father (Fig. 3). Following the American College of Medical Genetics, both the variants were classified as “pathogenetic” [5]. A chest x-ray showed no alterations in the mineralization of the ribs and sternum; nephrocalcinosis was ruled out by a bilateral kidney ultrasound. Eye counseling excluded corneal and conjunctival calcinosis and indirect signs of intracranial hypertension due to craniosynostosis. Blood tests and radiological investigations were also conducted on the child’s parents and resulted normal. Total serum ALP levels were within the normal range in the father (45 U/L) and in the mother (60 U/L for the mother). At the time of diagnosis, the mother was also pregnant (8th week of gestation). Consequently, genetic counseling and amniocentesis were performed, but the results excluded the presence of a pathogenic genotype in the developing fetus.

**Table 1:** Laboratory tests at presentation evidenced a severe deficiency of alkaline phosphatase (ALP) and elevated serum phosphorus and vitamin B6 values; other parameters were within normal range

Parameter	Value	Normal range
Red blood cells (10^6^/mm^3^)	5.01	3.7—5.3
Haemoglobin (g/dL)	12.3	11.5—13.5
White blood cells (10^3^/µL)	9.90	5—17.5
Platelets (10^3^/mm^3^)	358	300—700
Creatinine (mg/dL)	0.30	0.2—0.5
Sodium (mmol/L)	135	136—146
Potassium (mmol/L)	4.9	3.4—5.10
Clorum (mmol/L)	104	98—106
Calcium (mmol/L)	10.4	8.5—10.5
Phosphorum (mmol/L)	**7.13**	**4.5—6.5**
AST (UI/L)	36	15—60
ALT (UI/L)	20	8—20
TSH (µUI/ml)	2.81	0.25—4.5
FT4 (ng/dl)	1.26	0.70—1.70
PTH (pg/ml)	23.7	8.7—79.6
Vitamin D (ng/ml)	37.7	31—100
PLP (Vitamin B6) (µg/L)	**383**	**17—70**
ALP (UI/L)	**20**	**150—700**

Abbreviations: pyridoxal-5’-phosphate, PLP

During the follow-up, the baby also experienced the loss of three deciduous teeth at the age of 17 months.

At the age of 18 months, asfotase alpha was started at a dosage of 2 mg/kg for 3 times a week, with a normalization of serum phosphorus and ALP levels. After 6 months, a follow-up x-ray of the lower limbs revealed nearly complete disappearance of the area of rarefaction with osteolytic characteristics and significant improvement of the genu valgus, with a slight bending of the diaphysis of the tibias and fibulas. Subsequent radiographic examinations were conducted every 12 months. At the age of 7 years, a follow-up radiographic examination showed a “bowing” appearance of the tibial diaphysis without focal morphostructural changes in the examined bone segments (Fig. 2C). The patient is still taking subcutaneous asfotase alpha at a dosage of 2 mg/kg, 3 times a week. Moreover, the child did not develop any other skeletal, neurodevelopmental, dental, or auxological abnormalities.

## Discussion and conclusions

HPP is a rare genetic systemic disease characterized by a heterogeneous clinical expression and genetic background, with often serious long-term implications. Its prevalence ranges from 1:6370 to 1:1700 [2, 6]. To date, according to “The Genome Aggregation Database” (gnomAD), 287 nonsynonymous pathogenic or likely pathogenic *ALPL* variants and 247 variants of uncertain significance are known. HPP is considered a continuum of disease with five major metabolic conditions with different severity and age at onset: the perinatal form, the infantile form, the childhood form, the adult type, and odontohypophosphatasia [3]. The severity of the phenotype is directly related to the age at onset, with early-onset cases usually exhibiting more serious and multisystemic involvement. Perinatal HPP is evident at birth and is considered the most severe form, presenting with pyridoxine-dependent seizures, unexplained fever, hypoplastic lungs, periodic apnea, unmineralized bones, rickets, and premature death [3]. Infantile HPP has its onset before the age of 6 months, manifesting with rickets and skeletal deformities due to disrupted bone mineralization, weakness, increased intracranial pressure, hypercalcemia and hypercalciuria, renal involvement, and sometimes pyridoxine-dependent seizures [3]. Childhood HPP typically presents in children older than 6 months, with a more heterogeneous phenotype. Premature loss of deciduous teeth is observed in almost all patients, together with growth delay, muscle weakness, and skeletal deformities such as metaphyseal flaring, often resulting in joint enlargement [3]. Adult-onset HPP is typically the milder form, presenting with tooth loss, metatarsal stress fractures, osteomalacia, and recurrent arthromyalgia. Radiographic findings in children with HPP are represented by “tongues” of radiolucency due to reduced mineralization (tulip-shaped central metaphyseal lucencies are very typical), a progressive bending of the tibial and fibular diaphysis, and various skull and chest abnormalities [3, 7].

**Fig. 4:**
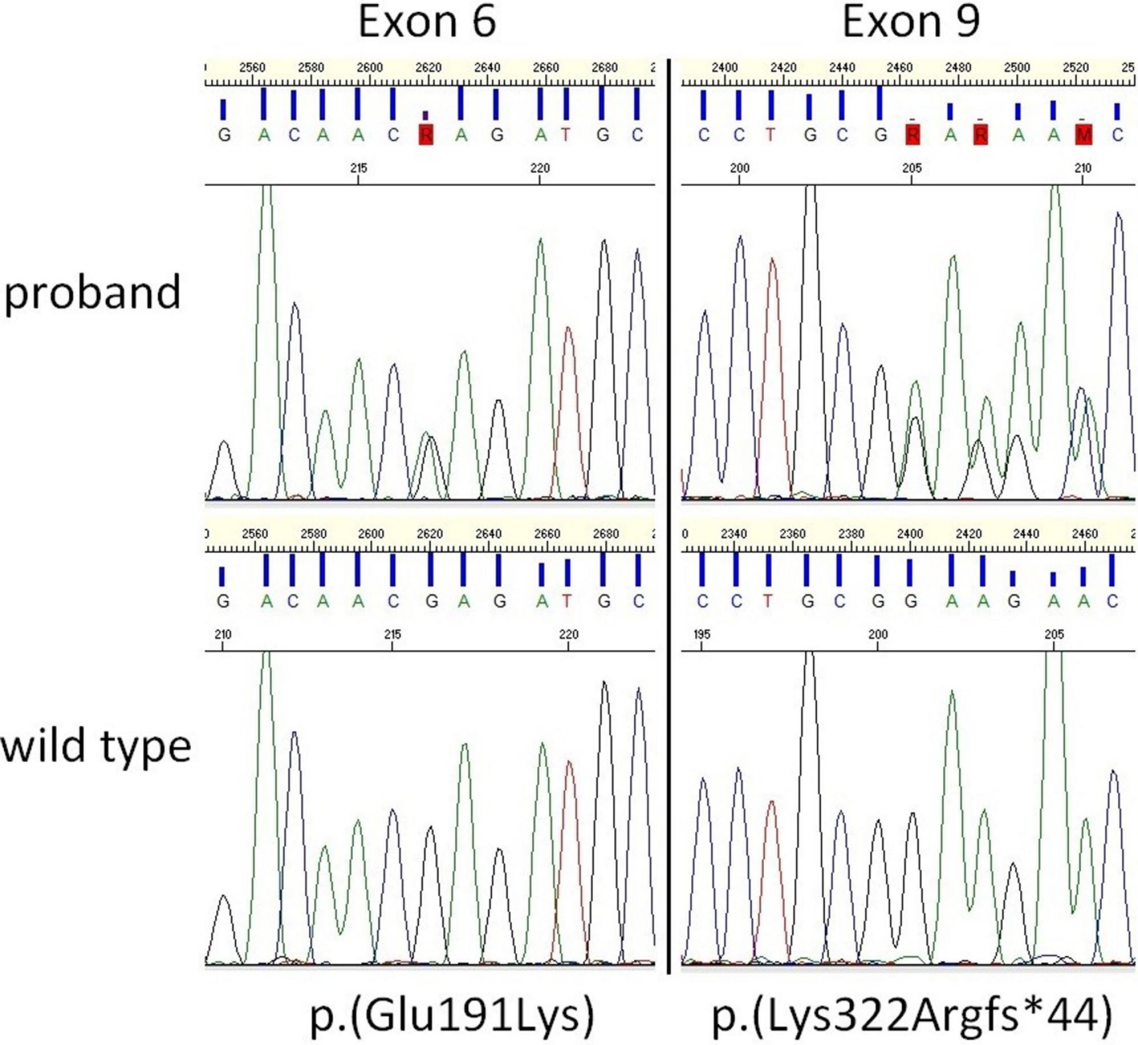
Diagram showing both the pathogenic variants in exons 6 and 9 as in the DNA of the proband (top) and on a wild type control DNA (bottom)

In this report, we describe a 15-month-old girl with two previously but individually described *ALPL* variants in compound heterozygosity: a missense change c.571G > A, p.(Glu191Lys) in exon 6 and a frameshift deletion c.963delG, p.(Lys322Argfs*44) in exon 9, resulting in childhood HPP [8]. According to the *ALPL* gene variant database (https://alplmutationdatabase.jku.at/), both the frameshift deletion c.963delG and the missense variant c.571G > A are classified as pathogenetic, as also described in clinical reports and functional testing (Fig. 4) [8, 9]. However, to the best of our knowledge, they have never been reported together in compound heterozygosity.

Our patient developed a childhood HPP with a severe clinical course, characterized by limping, severe and progressive skeletal deformities such as genu valgus, multiple osteolytic areas with osteopenia, and precocious deciduous tooth loss. Moreover, we documented elevated PLP levels, a condition that is commonly observed in childhood HPP and, by some authors, is considered a biochemical hallmark of HPP; however, this point has not been totally elucidated [4, 10]. Interestingly, the same variants were detected in the parents, who were, instead, completely asymptomatic.

The child was promptly treated with asfotase alpha (Strensiq^®^) with a quick improvement of clinical manifestations and radiological abnormalities. After a long-term follow-up of 6 years, no side effects occurred, and good tolerance was noted. Pediatricians should consider HPP as a possible diagnosis in children with a suggestive clinical presentation to make an early diagnosis and set the right, effective, and safe treatment.

## Data Availability

Data sharing is not applicable to this article as no datasets were generated or analyzed during the current study.

## Abbreviations

HPP: Hypophosphatasia
ALP: alkaline phosphatase
TNSALP: tissue-non-specific alkaline phosphatase
PLP: pyridoxal-5’-phosphate

## References

[1] Rush E, Brandi ML, Khan A, Ali DS, Al-Alwani H, Almonaei K, et al. Proposed diagnostic criteria for the diagnosis of hypophosphatasia in children and adolescents: results from the HPP International Working Group. Osteoporos Int. 2024;35:1–10. 37982855 10.1007/s00198-023-06843-2 PMC10786745

[2] Martínez-Heredia L, Muñoz-Torres M, Sanabria-de la Torre R, Jiménez-Ortas Á, Andújar-Vera F, González-Cejudo T, et al. Systemic effects of hypophosphatasia characterization of two novel variants in the ALPL gene. Front Endocrinol (Lausanne). 2023;14:1320516. 38234425 10.3389/fendo.2023.1320516 PMC10792043

[3] Whyte MP. Hypophosphatasia - aetiology, nosology, pathogenesis, diagnosis and treatment. Nat Rev Endocrinol. 2016;12:233–46. 26893260 10.1038/nrendo.2016.14

[4] Baroncelli GI, Carlucci G, Freri E, Giuca MR, Guarnieri V, Navarra G et al. The diagnosis of hypophosphatasia in children as a multidisciplinary effort: an expert opinion. J Endocrinol Invest. 2023. 10.1007/s40618-023-02199-w PMC10904512 37752373

[5] Richards S, Aziz N, Bale S, Bick D, Das S, Gastier-Foster J, et al. Standards and guidelines for the interpretation of sequence variants: a joint consensus recommendation of the American College of Medical Genetics and Genomics and the Association for Molecular Pathology. Genet Med. 2015;17:405–24. https://linkinghub.elsevier.com/retrieve/pii/S1098360021030318 10.1038/gim.2015.30 PMC4544753 25741868

[6] Mornet E, Yvard A, Taillandier A, Fauvert D, Simon-Bouy B. A molecular‐based estimation of the prevalence of hypophosphatasia in the European population. Ann Hum Genet. 2011;75:439–45. https://onlinelibrary.wiley.com/doi/10.1111/j.1469-1809.2011.00642.x 10.1111/j.1469-1809.2011.00642.x 21488855

[7] Mannes I, Rothenbuhler A, Merzoug V, Di Rocco F, Linglart A, Adamsbaum C. Imaging patterns in pediatric hypophosphatasia. Pediatr Radiol. 2022;52:998–1006. 10.1007/s00247-021-05232-3. 10.1007/s00247-021-05232-3 34854966

[8] Spentchian M, Merrien Y, Herasse M, Dobbie Z, Gläser D, Holder SE, et al. Severe hypophosphatasia: characterization of fifteen novel mutations in the ALPL gene. Hum Mutat. 2003;22:105–6. 12815606 10.1002/humu.9159

[9] del Angel G, Reynders J, Negron C, Steinbrecher T, Mornet E. Large-scale in vitro functional testing and novel variant scoring via protein modeling provide insights into alkaline phosphatase activity in hypophosphatasia. Hum Mutat. 2020;41:1250–62. https://www.ncbi.nlm.nih.gov/pmc/articles/PMC7317754/10.1002/humu.24010 PMC7317754 32160374

[10] Whyte MP, Zhang F, Wenkert D, Mack KE, Bijanki VN, Ericson KL, et al. Hypophosphatasia: vitamin B6 status of affected children and adults. Bone. 2022;154:116204. 34547524 10.1016/j.bone.2021.116204

